# Virtual occlusive artery in endovascular therapy for superficial femoral artery chronic total occlusion

**DOI:** 10.1093/ehjimp/qyae087

**Published:** 2024-08-20

**Authors:** Osamu Kurihara, Nobuaki Kobayashi, Masamichi Takano, Kuniya Asai

**Affiliations:** Cardiovascular Center, Nippon Medical School Chiba Hokusoh Hospital, 1715 Kamakari, Inzai, Chiba 270-1694, Japan; Cardiovascular Center, Nippon Medical School Chiba Hokusoh Hospital, 1715 Kamakari, Inzai, Chiba 270-1694, Japan; Cardiovascular Center, Nippon Medical School Chiba Hokusoh Hospital, 1715 Kamakari, Inzai, Chiba 270-1694, Japan; Department of Cardiovascular Medicine, Nippon Medical School, Tokyo, Japan

**Keywords:** computed tomography, chronic total occlusion, endovascular therapy, superficial, femoral artery

A 74-year-old man presented with severe claudication in the left leg, which did not respond to medical therapy. The ankle–brachial index (ABI) was abnormal (0.78; normal limit > 1.0). Contrast-enhanced computed tomography (CT) and angiography revealed total occlusion of the left superficial femoral artery (SFA; *Figure 1A*; Supplementary data online, *Video S1*). We created a 3D roadmap of a contrast-enhanced patent vessel and a virtual occlusive vessel from thin-slice CT data of the arterial phase using a SYNAPSE VINCENT computer workstation (FUJIFILM, Tokyo, Japan; *Figure 1B*). We fused the 3D roadmap with fluoroscopy in real-time using enhanced vessels with contrast media and bone shadows (*Figure 1C and D*; Supplementary data online, *Video S2*). We used Gladius® and Halberd® guidewires (Asahi Intecc Co., Aichi, Japan) and a Prominent® microcatheter (Tokai Medical Products Inc., Aichi, Japan) in an antegrade manner. We advanced the guidewire while aiming at the centre of the virtual occluded SFA on the 3D roadmap and successfully passed it in an antegrade manner (*Figure 1E*; Supplementary data online, *Video S3*). After pre-dilation of the occluded segments was performed, drug-eluting stents (7 × 150 mm, 7 × 40 mm; ELUVIA®; Boston Scientific, MA, USA) were deployed. After post-dilation of the stents, the final angiography revealed good stent expansion and sufficient flow to the left leg (Supplementary data online, *Video S4*). Consequently, the patient’s ABI improved to 1.12. Chronic total occlusion (CTO) intervention requires broad technical knowledge and experience. Our method visually assists the passage of guidewires within the CTO by accurately creating and depicting the occluded vessel. Real-time fusion of angiography and 3D roadmap, including virtual occlusive vessels constructed by CT, may facilitate and standardize the procedures for CTO lesions.

**Figure 1:**
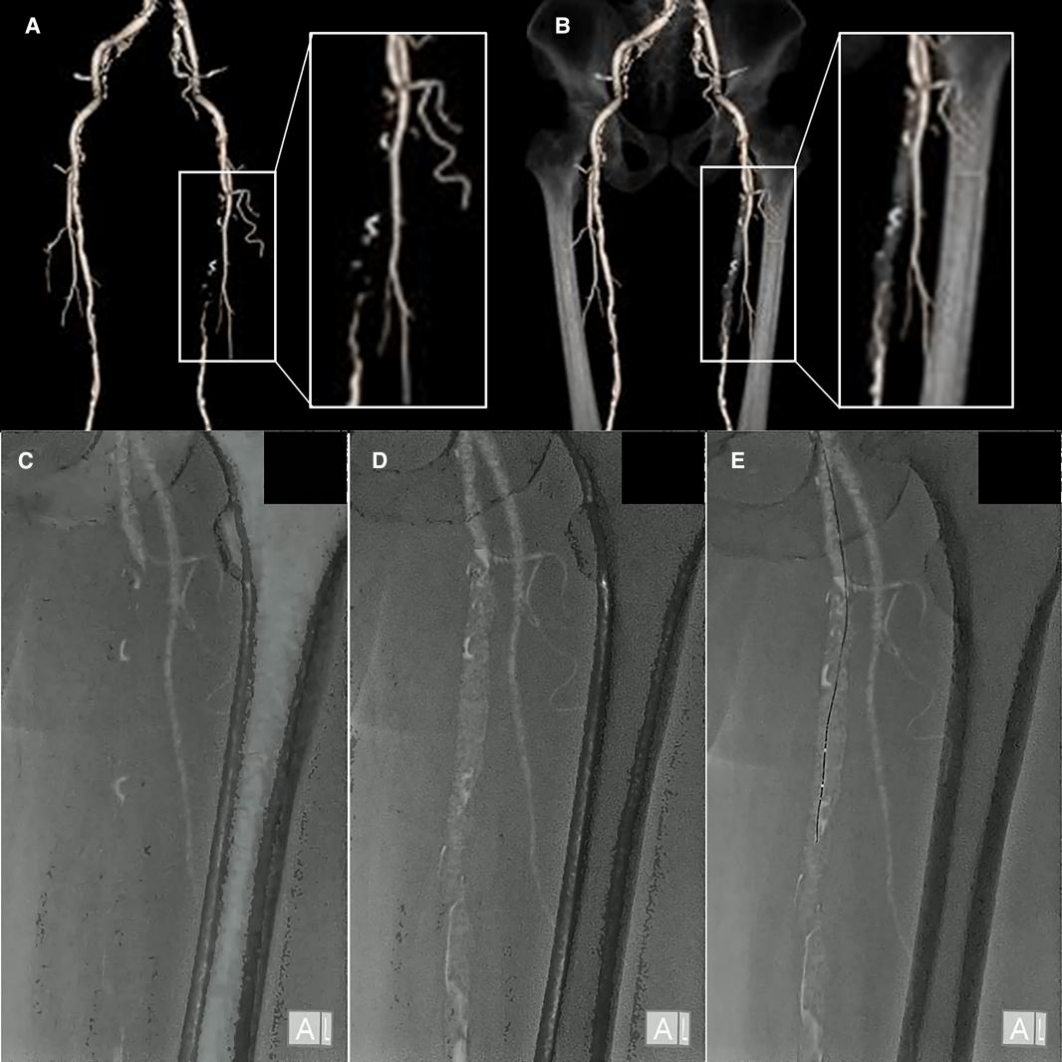
Contrast-enhanced computed tomography and fluoroscopy.

## Supplementary Material

qyae087_Supplementary_Data

## Supplementary data

**Funding:** None declared.

**Data availability:** No new data were generated or analysed in support of this article.

## Lead author biography

Osamu Kurihara 2007–2011: Fellow, Nippon Medical School Chiba Hokusoh Hospital. 2009–2018: Assistant professor, Cardiology Division, Nippon Medical School Chiba Hokusoh Hospital. 2018–2020: Research fellow, Cardiology Division, Massachusetts General Hospital, Harvard Medical School under the direction of Professor Ik-Kyung Jang. 2020–Present: Assistant professor, Cardiology Division, Nippon Medical School Chiba Hokusoh Hospital.

